# Genetically Modified Rabbits for Cardiovascular Research

**DOI:** 10.3389/fgene.2021.614379

**Published:** 2021-02-02

**Authors:** Jianglin Fan, Yanli Wang, Y. Eugene Chen

**Affiliations:** ^1^Department of Pathology, Xi'an Medical University, Xi'an, China; ^2^Department of Molecular Pathology, Faculty of Medicine, Graduate School of Interdisciplinary Research, University of Yamanashi, Yamanashi, Japan; ^3^School of Biotechnology and Health Sciences, Wuyi University, Jiangmen, China; ^4^Center for Advanced Models for Translational Sciences and Therapeutics, University of Michigan Medical Center, Ann Arbor, MI, United States

**Keywords:** atherosclerosis, CRISPR-Cas9, hypercholesterolemia, knock-out rabbits, transgenic rabbits

## Abstract

Rabbits are one of the most used experimental animals for investigating the mechanisms of human cardiovascular disease and lipid metabolism because they are phylogenetically closer to human than rodents (mice and rats). Cholesterol-fed wild-type rabbits were first used to study human atherosclerosis more than 100 years ago and are still playing an important role in cardiovascular research. Furthermore, transgenic rabbits generated by pronuclear microinjection provided another means to investigate many gene functions associated with human disease. Because of the lack of both rabbit embryonic stem cells and the genome information, for a long time, it has been a dream for scientists to obtain knockout rabbits generated by homologous recombination-based genomic manipulation as in mice. This obstacle has greatly hampered using genetically modified rabbits to disclose the molecular mechanisms of many human diseases. The advent of genome editing technologies has dramatically extended the applications of experimental animals including rabbits. In this review, we will update genetically modified rabbits, including transgenic, knock-out, and knock-in rabbits during the past decades regarding their use in cardiovascular research and point out the perspectives in future.

## Introduction

Rabbits were first used for disclosing the pathogenesis of human atherosclerosis a century ago. In 1908, a Russian physician, Alexander I. Ignatowski (1875–1955) fed rabbits with a diet supplemented with animal proteins (milk, meat, and eggs) and found that these rabbits developed pronounced aortic atherosclerosis.

Later, a Russian experimental pathologist, Nikolai N. Anichkov (or Anitschkow) (1885–1964) further demonstrated that it was dietary cholesterol rather than proteins that play the critical role in the pathogenesis of atherosclerosis in rabbits and proposed a causal role of cholesterol in the development of atherosclerosis (Fan et al., 2015). Now, a consensus has been widely hold in this field that, in both humans and experimental animals, high levels of plasma cholesterol carried by apolipoprotein (apo)-B-containing particles such as low density lipoproteins (LDL) initiate the development of atherosclerosis (Steinberg, 2004). These pioneering studies derived from rabbit experiments not only provided the first evidence but also established a theory basis of the “lipid hypothesis” of atherosclerosis (Steinberg, 2004). Since then, cholesterol-fed rabbits along with Watanabe heritable hyperlipidemic (WHHL) rabbits, a mutant rabbit with genetic deficiency of LDL receptor functions, have been extensively used to elucidate multiple facets of the pathophysiology of human atherosclerosis, leading to the discovery of the LDL receptor functions in familial hypercholesterolemia (Goldstein et al., 1983) and the development of the most-prescribed lipid-lowering drug, statin (Brown and Goldstein, 2004). On the other hand, transgenic rabbits with overexpression of various genes were generated from early 90's and served as an alternative tool for investigating the gene functions in cardiovascular disease. Moreover, recent genome editing technology has provided enormous opportunities to create knock-out (KO) and knock-in (KI) rabbits. Important roles of rabbits in studying human atherosclerosis have been extensively reviewed in the previous reviews (Fan et al., 1999a, 2015, 2018; Fan and Watanabe, 2000, 2003). In this review, we will focus on genetically modified rabbits for their applications in cardiovascular research.

## “Naturally” Genetically Modified Rabbits

Spontaneous mutations in rabbits can be found accidentally and they can be used in controlling the coat color for commercial purposes such as tyrosinase and the melanocortin 1 receptor (Aigner et al., 2000; Xiao et al., 2019). However, some spontaneous mutations in rabbits can cause a pronounced phenotype that can mimic human diseases, such as Watanabe heritable hyperlipidemic (WHHL) rabbits (Watanabe, 1980), St. Thomas hyperlipidemic rabbits (Laville et al., 1987; Seddon et al., 1987) and complement 6 deficient rabbits (Rother, 1986; Liu et al., 2007a). WHHL rabbits were originally established by Dr. Yoshio Watanabe (1927–2008) at Kobe University, Japan, through serial inbreeding (Watanabe, 1980). Homozygous WHHL rabbits exhibit spontaneous hypercholesterolemia characterized by high levels of LDLs and severe atherosclerosis and often serve as a human familial hypercholesterolemia model (Watanabe et al., 1985). Genetic analysis revealed that WHHL rabbits have defective LDL receptor functions due to a deletion of 12 nucleotides in exon 4 of the LDL receptor gene, which leads to a 4-amino acid deletion in the cysteine-rich ligand-binding domain of the LDL receptor protein (Yamamoto et al., 1986). LDL receptor mutations can be easily detected by PCR analysis (Sun et al., 2002a); however, high levels of plasma LDL-cholesterol are the major manifestation observed in homozygous WHHL rabbits. In addition to hyperlipidemia and aortic atherosclerosis, some WHHL rabbits (later designated as WHHL-MI) show coronary atherosclerosis and myocardial infarction (Shiomi et al., 2003; Shiomi and Fan, 2008). Using WHHL rabbits, Tomoike et al. further developed a subline of WHHL designated a hereditary hypertriglyceridemic rabbit after selected in-breeding. This model exhibited postprandial hypertriglyceridemia along with insulin resistance and visceral obesity although polygenetic loci for these pathophysiogical changes have not been determined (Kawai et al., 2006). In addition to WHHL rabbits, the St. Thomas hospital hyperlipidemic rabbits were developed by La Ville et al. in London (Laville et al., 1987; Seddon et al., 1987). Different from WHHL rabbits which have high plasma LDL levels due to LDL receptor dysfunctions, the St. Thomas hospital hyperlipidemic rabbits showed high levels of very low density lipoproteins (VLDL) and intermediate density lipoproteins (IDL), and LDL, thus this rabbit model resembles human familial combined hyperlipidemia. Elevated plasma cholesterol levels in these rabbits were caused by overproduction of these apo-B-containing lipoproteins in the liver although the genetic mutations responsible for hyperlipidemia have not been examined in details. There is a complement-6 (C6) deficient rabbit originally reported by Rother in 1986 (Rother, 1986). C6 deficiency in these rabbits arises from a single gene defect and is not known to be associated with other genetic abnormalities. In spite of this, C6 deficient rabbits are protective against cholesterol diet-induced atherosclerosis (Schmiedt et al., 1998).

## Transgenic Rabbits

Because spontaneous mutant rabbits with obvious phenotypes resembling human disease phenotypes are rare and accidently discovered by experimental animal staff, it is necessary to make genetically modified rabbits according to one's own research purposes. The technology for producing transgenic (Tg) rabbits was almost concurrently reported by German (Brem et al., 1985) and US (Hammer et al., 1985) groups in 1985, but the actual use of Tg rabbit technology as an experimental tool in the field of cardiovascular diseases was not realized until 1994 when John Taylor's laboratory at the Gladstone Institute of Cardiovascular Disease in San Francisco created the first Tg rabbit expressing human hepatic lipase (Fan et al., 1994). Later on, they also produced Tg rabbits expressing human apoB-100 (Fan et al., 1995), apoE (Huang et al., 1997; Fan et al., 1998), and apoB mRNA editing protein (Yamanaka et al., 1995). Until now, more than 20 kinds of Tg rabbits expressing different genes that are involved in lipid metabolism and atherosclerosis have been reported and studies using these Tg rabbits have provided considerable insights into the molecular mechanisms of these gene functions in lipoprotein metabolism and atherosclerosis (Fan and Watanabe, 2003; Peng, 2012; Fan et al., 2015). The transgenes expressed in Tg rabbits for the study of lipoprotein metabolism and atherosclerosis can generally be classified into three categories: (1) those proteins that constitute lipoprotein structures such as apo(a) (Rouy et al., 1998; Fan et al., 1999b), apoAI (Duverger et al., 1996a), apoAII (Koike et al., 2009a; Wang et al., 2013), apoB-100 (Fan et al., 1995), apoCIII (Ding et al., 2011), and apoE (Huang et al., 1997; Fan et al., 1998); (2) those enzymes or transfer proteins that participate in the lipid metabolism such as hepatic lipase (Fan et al., 1994), lipoprotein lipase (Fan et al., 2001a), phospholipid transfer protein (Masson et al., 2011), apoB-100 mRNA editing enzyme catalytic polypeptide protein (Yamanaka et al., 1995), lecithin:cholesterol acyltransferase (Hoeg et al., 1996), endothelial lipase (Wang et al., 2017); and (3) those proteins that may exert some functions on the arterial wall cells which participate in the pathogenesis of atherosclerosis including matrix metalloproteinase-1,9,12 (Liang et al., 2006; Niimi et al., 2019; Chen et al., 2020), 15-lipoxygenase (Shen et al., 1996), C-reactive protein (Koike et al., 2009b), and vascular endothelial growth factor (Kitajima et al., 2005) (Table 1). In addition, Tg rabbits have also been used for the investigation of human heart diseases, including LQT syndrome (Brunner et al., 2008), hypertrophic cardiomyopathy (Marian et al., 1999) and tachycardia-induced cardiomyopathy (Suzuki et al., 2009). This is because, in comparison with mice and rats, the rabbit heart is similar to that of humans in both structure and function (Bers, 2002; Marian, 2006; Pogwizd and Bers, 2008). For example, like human heart in which β-myosin heavy chain (β-MyHC) accounts for 90% of total myofibrillar myosin, rabbit heart is composed of 80% β-MyCH which is different from the mouse heart predominated by 95% α-MyHC (Marian, 2005; Bosze et al., 2016). Tg rabbits can be generated by microinjecting a transgenic DNA construct into the pronuclei of fertilized embryos (Fan et al., 1999a; Kitajima et al., 2003). The transgenic constructs are typically composed of the transgene (either cDNA or genomic DNA) under the control of a tissue-specific promoter such as liver- and macrophage-specific promoter. In addition to the pronuclear microinjection method, other methods such as sperm vector (Wang et al., 2003; Li et al., 2006, 2010; Shen et al., 2006), ICSI-mediated transgenesis (Li et al., 2010; Zhang et al., 2016), somatic cell nuclear transfer (SCNT) (Li et al., 2009) or chimeric SCNT (Matsuda et al., 2002; Skrzyszowska et al., 2006), lentiviral vectors (Hiripi et al., 2010), transposon-mediated transgenesis (Katter et al., 2013; Ivics et al., 2014), and novel genome editing technology (Song J. et al., 2016; Yang et al., 2016; Li et al., 2019) have been reported to produce Tg rabbits. In spite of this, the pronuclear microinjection is still the most common method even though transgene integration rate is low.

**Table 1:** Transgenic rabbits for the study of human lipoproteins and atherosclerosis.

**Genes**	**Expression cells**	**Major phenotypes**	**References**
**Apolipoproteins (apo)**			
Apo(a)	Liver	Atherogenic	Rouy et al., 1998; Fan et al., 2001b; Ichikawa et al., 2002; Sun et al., 2002b; Kitajima et al., 2007
Apo(a) and apoB	Liver	Not determined	Rouy et al., 1998
ApoA-I	Liver	Athero-protective	Duverger et al., 1996a,b
ApoA-II	Liver	Athero-protective	Koike et al., 2009a; Wang et al., 2013
ApoA-I/C-III/A-IV	Liver and intestine	No effect on atherosclerosis	Recalde et al., 2004
ApoB-100	Liver	LDL↑, HDL↓	Fan et al., 1995
ApoCIII	Liver	VLDL↑	Ding et al., 2011
ApoE2	Liver	Atherogenic	Huang et al., 1997
ApoE3	Liver	Atherogenic	Fan et al., 1998; Huang et al., 1999
**Enzymes or transfer proteins**			
APOPEC1	Liver	LDL↓, liver carcinoma	Yamanaka et al., 1995
APOPEC1	Knockdown by RNAi	Lean	Jolivet et al., 2014
CETP	Liver	HDL↓	Gao et al., 2017
Endothelial lipase	Liver	Atheroprotective	Wang et al., 2017; Yan et al., 2020b
Hepatic lipase	Liver	Athero-protective	Fan et al., 1994
LCAT	Liver	Athero-protective	Hoeg et al., 1996
Lipoprotein lipase	Universal	Athero-protective	Fan et al., 2001a
PLTP	Universal	Atherogenic	Masson et al., 2011
**Vascular cell factors**			
C-reactive protein	Liver	Thrombogenic	Matsuda et al., 2011
Lipoprotein lipase	Macrophage	Atherogenic	Ichikawa et al., 2005
15-lypooxygenase	Macrophage	Athero-protective	Shen et al., 1996
MMP-1	Macrophage	Aortic aneurysm↑	Niimi et al., 2019
MMP-9	Macrophage	Vascular calcification	Chen et al., 2020
MMP-12	Macrophage	Atherogenic	Liang et al., 2006; Yamada et al., 2008
Urotensin II	Macrophage	Atherogenic	Zhao et al., 2015
VEGF	Liver	Hemangiomas and impaired glomerular functions	Kitajima et al., 2005; Liu et al., 2007b

*APOPEC1, apolipoprotein B mRNA editing enzyme, catalytic polypeptide 1; CETP, cholesteryl ester transfer protein; LCAT, lecithin:cholesterol acyltransferase; PLTP, phospholipid transfer protein; MMP, matrix metalloproteinase; VEGF, vascular endothelial cell growth factor. ↑, increase; ↓, decrease*.

## Rabbit Embryonic Stem Cells and Genome Information

Because of the lack of both rabbit embryonic stem (ES) cells and the genome information, it has been considered impossible to create KO rabbits by homologous recombination-based genomic modification as to generate KO mice. Unavailability of KO rabbits also constitutes another obstacle that hampers researchers to study loss-of-functions of genes in rabbits. We strived to use somatic cell nuclear transfer technique to generate KO rabbits after Chesne et al. reported the first cloned rabbit about 17 years ago (Chesne et al., 2002). However, after enormous attempts, we got to the conclusion that the production of KO rabbits by somatic cell nuclear transfer is far remote from reality. As a research tool, nuclear transfer technique is unworkable owing to the extraordinarily low efficiency of gene transfer into somatic cells and the possibility in generating cloned rabbits (Song J. et al., 2020). Many groups reported that they could obtain rabbit ES-like cells, but none of these so-called ES-like cells have been proved to be able to generate chimera rabbits (Fan et al., 2015). Rabbit genome has long been an empty area mainly because of budget insufficiency and narrow research communities. In 2014, Carneiro et al. successfully reported a high-quality reference genome using the European rabbit with references to domestication and speciation (Carneiro et al., 2014a,b). Almost at the same period, we along with researchers from the US, Japan and China organized an International Rabbit Genome Sequencing Project Consortium aiming at implementing more extensive whole-genome sequencing of three kinds of common laboratory rabbits: Japanese white rabbits, New Zealand white rabbits and WHHL rabbits. In addition, we performed deep transcriptome sequencing of the aortas, livers, hearts, and kidneys of cholesterol-fed and WHHL rabbits (Wang et al., 2016). After a 2-year collaborative work, we were able to completed whole-genome sequencing of 10 male rabbits for each line with coverage of 13x for each individual after alignment to the reference genome. With the successful completion of rabbit genome sequencing (Carneiro et al., 2014a,b; Wang et al., 2016), researchers now can easily not only design PCR primers to study gene expression in rabbits but also to generate KO rabbits using genome editing techniques as described below. Rabbit genome information is now available from the NCBI database and a comprehensive rabbit transcriptome information established by the Chinese Academy of Sciences in Shanghai (Zhou et al., 2018) is also available at http://www.picb.ac.cn/RabGTD/.

## Knock-Out and Knock-In Rabbits by Genome Editing Techniques

In the past decade, the emergence of three powerful genome editing technologies has dramatically enhanced the application of genetically modified rabbits (Song J. et al., 2020). The first one is the zinc finger nuclease (ZFN)-mediated genome editing method by which KO rats were successfully created in 2009 (Geurts et al., 2009). Two years later after the birth of KO rats, Flisikowska et al. generated the first immunoglobulin KO rabbits in an attempt to produce humanized antibodies (Flisikowska et al., 2011). Almost at the same time, we successfully created apoCIII KO rabbits with ZFN-mediated genome editing technology (Yang et al., 2013). ZFNs are engineered DNA-cleaving enzymes made by fusing a tailor-made DNA-binding domain to the DNA cleavage domain of Fok1, a type II restriction enzyme. ZFNs generate site-specific double-strand breaks in the DNA at researcher-assigned sites, thus resulting in targeted modification of the genome. However, while ZFNs were not extensively applied in this field, the second generation of the genome editing tool, transcription activator-like effector nucleases (TALEN) were shown up to make the first KO rats in 2011 (Tesson et al., 2011). TALENs are considered much simpler to design and assemble than ZFNs. The DNA binding domain in TALENs was derived from *Xanthomonas spp. Bacteria* (Christian et al., 2010; Miller et al., 2011). While TALENs utilize the same Fok I endonuclease domain as ZFNs, its DNA binding domain contains a repeated highly conserved 33–34 amino acid sequence with divergent 12th and 13th amino acids which called Repeat Variable Diresidue (RVD) for recognizing one specific nucleotide, for example, NN for guanine, NI for adenine, HD for cytosine, and NG for thymine. This direct relationship between amino acid sequence and DNA recognition has made engineering sequence specific binding domains much easier than ZFNs (Boch et al., 2009; Moscou and Bogdanove, 2009). Using TALEN technology, Lai's laboratory at GIBH, immediately generated two kinds of KO rabbits: an immunodeficent KO rabbit with deficiency of Rag1 and Rag2 genes (Song et al., 2013) and fumarylacetoacetate hydrolase deficient rabbits (Li et al., 2017), which mimics human genetic disease tyrosinemia type I, an autosomal recessive disorder caused by mutations in the both copies of the gene encoding the enzyme. Although TALENs are considered superior to ZFNs in terms of fewer off-target effects, easy design and production, it was soon replaced by the CRISPR-Cas9 based genome editing technology, which is even more rapid and modular than the TALEN platform. Cas9 is an endonuclease playing a protective role against foreign nucleic acids in the adaptive immune system in bacteria. The feature of bacterial CRISPR immune system is that genetic materials taken up from previous invasive elements are expressed in crRNA, which could direct the Cas9 endonuclease to cut foreign DNA elements containing the same sequences (Jinek et al., 2012). Therefore, the CRISPR/Cas9 system has been remolded from bacterial immune system to the genome editing tool, using a designed RNA to guide Cas9 nuclease to the specific DNA sequence (Hsu et al., 2014). Binding of Cas9 nuclease on a specific protospacer adjacent motif (PAM) sequence on the genome (NGG for spCas9) will unwind the adjacent sequence, allowing the RNA:DNA pairing, which activates the nuclease domains in Cas9 to cut DNA and make double-strand breaks. While ZFNs and TALENs rely on protein-DNA recognition, which is less predictable for design and more labor and time consuming for assembly, the CRISPR/Cas9 system relies on the RNA–DNA recognition, which is much simpler and more predictable.

Because CRISPR-Cas9 technique is so efficient and powerful, it was quickly adopted to generate KO rabbits. In this respect, Chen's laboratory at the University of Michigan first established a number of KO rabbits aiming at studying human cardiovascular disease (Yang et al., 2014) and then KO rabbit boom started. Lai's laboratory at GIBH and Li's laboratory at Jilin University made more than 30 KO rabbits using CRISPR-Cas9 along with base-editing (Liu et al., 2018) and CRISPR/Cpf1 (Wu et al., 2018). Most KO rabbits were created in attempt to recapitulate human genetic or congenital disorders or immunodeficient rabbits (Song J. et al., 2017, 2018) as shown in Table 2. In addition, this technique has been tried to target the tyrosinase gene to modify rabbit coat colors (Honda et al., 2015; Song Y. N. et al., 2016, 2017, 2018). The standard protocol for generation of KO rabbits using CRISPR-Cas9 has been recently published (Yang et al., 2019). The techniques have been further refined (Liu et al., 2018, 2020) so we can predict that in the next few years, more and more KO or KI rabbits will be made using this technology. Here we will briefly review some valuable KO rabbits created recently to discuss their usefulness in disclosing the molecular mechanisms of atherosclerosis.

**Table 2:** Human congenital disease models of KO rabbits recently created by CRISPR-Cas9 or TALEN.

**Human diseases**	**Targeted genes**	**Major phenotypes**	**References**
**Congenital cataracts**	αA-Crystallin	Cataracts, microphthalmia, obscurity	Yuan et al., 2017
	GJ48	Microphthalmia, small lens size, and cataracts	Yuan et al., 2016
**Muscular dystrophy/hypertrophy**	ANO5	Muscular dystrophy with increased serum creatine kinase	Sui et al., 2018a
	DMD	Impaired physical activity, elevated serum creatine kinase	Sui et al., 2018b
	Myostatin	Hyperplasia or hypertrophy of muscle	Lv et al., 2016 (base editing)
**Metabolic diseases**			
	ATP7B	Wilson disease, Death at 3 mon	Jiang et al., 2018 (Precision point mutation)
	Dentin matrix protein 1	Mineralization defects	Liu et al., 2019
	GADD45G	Congenital defects cleft palate	Lu et al., 2019
	Glucokinase	Maturity-onset diabetes of the young 2 (MODY2)	Song Y. et al., 2020
	Fumarylacetoacetate hydroxylase	Hereditary tyrosinemia type 1	Li et al., 2017 (TALEN)
	HOXC13	Hair and nail ectodermal dysplasia	Deng et al., 2019
**Syndromes**			
	FBN1	Marfanoid-progeroid-lipodystrophy syndrome	Chen et al., 2018
	SRY	Sex reversal syndromes and hermaphroditism syndromes	Song Y. et al., 2017, 2018
	LMNA	Premature aging syndrome	

## ApoCIII KO Rabbits

ApoCIII is a major component of plasma chylomicrons and VLDLs, and is a minor component of high density lipoproteins (HDLs) and was first reported by Brown et al. 50 years ago (Brown et al., 1969). It is generally believed that physiological functions of apoCIII is to mediate the triglyceride(TG)-rich lipoprotein metabolism thereby maintaining the plasma TG homeostasis and high plasma levels of apoCIII are positively associated with plasma TG and increases the risk of ischemic heart disease (Huff and Hegele, 2013; Norata et al., 2015; Ramms and Gordts, 2018). However, for a long time, it is not clear whether apoCIII was directly involved in the pathogenesis of atherosclerosis because mouse models failed to provide a clear answer (Yan et al., 2020a). Yang et al. first generated apoCIII KO rabbits using ZNF (Yang et al., 2013) and after several years efforts to breed enough numbers of homozygous apoCIII KO rabbits, we were able to examine the hypothesis whether apoCIII may participate in atherosclerosis. Recently, we have shown that that genetic deletion of the apoCIII gene in KO rabbits significantly accelerates catabolism of TG-rich lipoproteins in the liver and apoCIII deficiency leads to the resistance of KO rabbits to a cholesterol diet-induced hyperlipidemia and inhibits atherosclerosis (Yan et al., 2020a). These results indicate that therapeutic inhibition of apoCIII expression may become a novel strategy for the treatment of hyperlipidemia and atherosclerosis.

## ApoE KO Rabbits

ApoE is a ligand for both LDL receptor and LRP and plays an important role in the catabolism of remnant lipoproteins in the liver and genetic deficiency of apoE is a cause of human type III hyperlipoproteinemia (Mahley, 1988; Mahley et al., 1999). Deletion of apoE in mice even on a normal chow diet exhibited hyperlipidemia along with spontaneous aortic atherosclerosis (Plump et al., 1992; Zhang et al., 1992). ApoE KO rabbits were produced at University of Michigan using CRISPR-Cas9 (Yang et al., 2014) and Sage Company using ZFN (Ji et al., 2015), respectively. Even though different techniques were adopted, apoE KO rabbits generated by these two methods exhibit the same phenotypes (Niimi et al., 2016). Homozygous apoE KO rabbits on a normal diet only showed mild hyperlipidemia and their plasma total cholesterol levels reached ~200 mg/dL, similar to human type III hyperlipoproteinemia patients, whose cholesterol levels are elevated to 300~350 mg/dL (Mahley et al., 1999). Because plasma levels of cholesterol in apoE KO rabbits on a normal diet are not high to be atherogenic, there are not spontaneous atherosclerosis, which is different from apoE KO mice. However, when apoE KO rabbits were fed a cholesterol diet, they developed more prominent hypercholesterolemia than WT rabbits, which is basically caused by the remarkable accumulation of intestinally-derived remnant lipoproteins, β-VLDLs (Niimi et al., 2016). Recently, we found that apoE KO rabbits are highly susceptible to a cholesterol diet-induced atherosclerosis. Therefore, apoE KO rabbits will serve as a new model for human hyperlipidemia.

## LDL Receptor KO Rabbits

In humans, genetic deficiency of LDL receptor functions causes severe hypercholesterolemia and atherosclerosis at early ages, called familial hypercholesterolemia (FH). FH is an autosomal dominant genetic disorder characterized by elevated plasma LDL levels due to LDL receptor dysfunctions (Soutar and Naoumova, 2007). Two laboratories have successfully generated LDL receptor KO rabbits using CRISPR-Cas9 (Yang et al., 2014; Lu et al., 2018). Similar to human FH, homozygous LDL receptor KO rabbits develop spontaneous hypercholesterolemia and atherosclerosis (Lu et al., 2018). Therefore, like WHHL rabbits, LDL receptor KO rabbits can be used for the study of human FH.

## Cholesteryl Ester Transfer Protein KO Rabbits

Cholesteryl ester transfer protein (CETP) is a glycoprotein that transfers plasma lipids between HDLs and apoB-containing particles therefore plays an important role in lipoprotein metabolism. However, it is not known whether inhibition of CETP activity can prevent cardiovascular disease because four CETP inhibitors (torcetrapib, dalcetrapib, evacetrapib, and anacetrapib) failed to prove their efficacy in terms of reduction of cardiovascular risk by clinical trials(https://en.wikipedia.org/wiki/CETP_inhibitor). Since CETP is genetically absent in rodents (mice and rats) and pigs, rabbits are considered the best model for investigation of CETP functions because rabbits have high levels of CETP in the plasma as humans. Taking this advantage, Zhang et al. created CETP KO rabbits and found that CETP KO rabbits showed higher plasma levels of HDL-cholesterol (Zhang et al., 2017). When fed a cholesterol-rich diet, CETP KO rabbits still exhibited higher HDL-cholesterol levels accompanied by lower total cholesterol levels than wild-type (WT) rabbits (Zhang et al., 2017). CETP KO rabbits had significant less atherosclerosis in both aorta and coronary arteries than WT rabbits (Zhang et al., 2017). These results indicate that genetic ablation of CETP gene inhibits the development of atherosclerosis in cholesterol-fed rabbits.

## ApoAII KI Rabbits

ApoAII is the second major apolipoproteins in HDLs. However, its physiological functions are largely unknown compared with apoAI. Interestingly, WT rabbits are genetically deficient in apoAII so their HDLs only contain apoAI. This unique feature makes WT rabbits as a “natural” apoAII KO model. We first made Tg rabbits expressing human apoAII gene and found that hepatic expression of human apoAII inhibits cholesterol diet-induced atherosclerosis (Wang et al., 2013). To examine the apoAII specific functions in the absence of apoAI, we further replaced the rabbit endogenous apoAI with human apoAII gene through knock-in (KI) using TALEN technology (Koike et al., 2021). In this way, apoAII KI rabbits expressed exclusively human apoAII without apoAI in HDL particles, which enables us to compare the net functions of apoAI-only-HDLs in WT rabbits with apoAII-only-HDL in KI rabbits in terms of HDL metabolism and atherosclerosis. In the latest study, we found that apoAII KI rabbits showed consistently lower TG and higher HDL-cholesterol levels and developed significantly less aortic atherosclerosis on a cholesterol diet (Koike et al., 2021).

## Considerations and Future Perspectives

Although genetically modified rabbits are an important experimental model in cardiovascular research, they should not be simply used as a substitute of mice and rats, as discussed above. Because rabbits are more expensive, require larger space, and need more time to breed compared with mice, the generation of genetically modified rabbits should be carefully planned to solve those specific problems that cannot be well-examined in other experimental animal models, such as the development of lipid-lowering drugs (Niimi et al., 2020). However, off-target effects in these animals remain a concern as the genome editing is extremely productive and efficient. So far, almost all studies claimed that off-targets in genetically modified rabbits through genome editing are either none or negligible as comprehensively discussed in the recent review (Song J. et al., 2020); nevertheless, there is a need to performed careful genotyping, including sequencing, and expression validation of genetically modified rabbit models. It can be expected that more and more genetically modified rabbits will be made and used in a variety of medical sciences which will certainly expand our knowledge to explore new mechanisms of human diseases. Genome editing technique may eventually replace the pronuclear microinjection for the generation of Tg rabbits. However, complicated gene manipulation in rabbits, such as conditional KO in an organ- or cell-specific and time-controlled manner using the Cre/LoxP system is still lacking, thus it will be absolutely necessary to build such a platform in future. Finally, the preservation of valuable strains of genetically modified rabbits is an urgent task with increased number of rabbit models produced. In this aspect, various procedures for cryopreservation of rabbit sperm (Vicente and Viudes-de-Castro, 1996; Dalimata and Graham, 1997; Nishijima et al., 2015) and embryos (al-Hasani et al., 1992; Kasai et al., 1992; Marco-Jimenez et al., 2016) have been reported but have not been standardized. In the future, it may be necessary to establish an international rabbit bio-resource center or sperm and embryo bank to stock and share valuable rabbit models worldwide.

## Author Contributions

JF, YW, and YC wrote the manuscript. All authors contributed to the article and approved the submitted version.

## Conflict of Interest

The authors declare that the research was conducted in the absence of any commercial or financial relationships that could be construed as a potential conflict of interest.

## Abbreviations

Apo: Apolipoprotein
Cas9: CRISPR-associated (Cas) protein 9
CETP: Cholesteryl ester transfer protein
CRISPR: Clustered regularly interspaced short palindromic repeat
FH: Familial hypercholesterolemia
HDL: High density lipoproteins
IDL: Intermediate density lipoproteins
LDL: Low density lipoproteins
KI: Knock-in
KO: Knock-out
TALEN: Transcription activator-like effector nuclease
Tg: Transgenic
VLDL: Very low density lipoprotein
WHHL: Watanabe heritable hyperlipidemic
ZFN: Zinc finger nuclease.
